# Anal human papillomavirus infection in HIV-positive men and women at two opportunistic infections clinics in Harare, Zimbabwe

**DOI:** 10.1186/s12889-018-6170-6

**Published:** 2018-11-14

**Authors:** Simbarashe Chinyowa, Joel M. Palefsky, Zvavahera M. Chirenje, Rudo Makunike-Mutasa, Marshall Munjoma, Godfrey I. Muguti

**Affiliations:** 1 Department of Surgery, University of Zimbabwe College of Health Sciences, P.O. Box A178 Avondale, Harare, Zimbabwe; 2 Department of Medicine, University of California, San Francisco, USA; 3 Department of Gynaecology, University of Zimbabwe College of Health Sciences, Harare, Zimbabwe; 4 Department of Histopathology, University of Zimbabwe College of Health Sciences, Harare, Zimbabwe

**Keywords:** Anal, Human papillomavirus, HIV, Men, Women, Africa

## Abstract

**Background:**

HIV-infected individuals are at increased risk of anal cancer; in the majority of cases this is linked to human papillomavirus (HPV) infection. Anal cancer screening is not routinely offered in Zimbabwe.

**Methods:**

A cross-sectional study was performed on 152 patients (88 females; 64 males) attending Opportunistic Infection Clinics at 2 tertiary hospitals between November 2014 and June 2015. Demographic data, immunological parameters and behavioural characteristics were collected. An anal swab was collected from each patient for HPV genotype testing. HPV testing was performed using MY09/MY11 PCR, followed by typing using the dot blot method.

**Results:**

The mean age was 39.6 years (range, 18–69 years). Median CD4 count was 375 cells/μL. 96% were on antiretroviral therapy. Only one patient identified as a man who has sex with men. Of 122 samples tested for HPV, 54 were positive (44%). HPV was three times more common in females (60%) than males (20%). Being HPV-positive was associated with history of perianal warts, history of cervical intraepithelial neoplasia and having more than ten lifetime sexual partners. The most commonly detected high-risk HPV genotypes were HPV-58 (13%), HPV-31 (11%) and HPV-16 (9%). Nine patients harboured multiple high-risk HPV types. The two most commonly detected low-risk genotypes were HPV-11 (17%) and HPV-53 (11%).

**Conclusion:**

Overall anal HPV prevalence was 44% in this mostly heterosexual HIV-positive population. Oncogenic HPV types accounted for almost half of infections, supporting the need for surveillance of anal cancer in this population.

## Introduction

Anal cancer is rare in the general population but is significantly more prevalent in high-risk groups such as men who have sex with men (MSM) and those infected with human immunodeficiency virus (HIV). In up to 90% of cases anal cancer is linked to human papillomavirus (HPV) infection [1]. HIV predisposes an individual to multiple and often persistent anal HPV infections. There has been a downward trend in HIV prevalence in Zimbabwe thanks to aggressive efforts to manage the epidemic. However, the overall burden of HIV in the general population is still high, at 13% [2].

A substantial amount of data are now available from studies on anal HPV infections and consequent disease in MSM. There are numerous HPV genotypes and anal cancer in this population is most strongly linked to persistent HPV-16 and HPV-18 infection [3]. However data from other high-risk groups, in particular, African heterosexual males and females are relatively scant [4].The patterns of HPV infection, HPV clearance or progression to anal squamous intraepithelial lesions and cancer in this latter group are yet to be fully elucidated.

Anecdotal observation and crude cancer registry data suggest an upward trend in the number of cases of anal cancer in Zimbabwe in the past few years. Anal cancer screening is not routinely offered in Zimbabwe. Against this backdrop we sought to establish the prevalence of anal HPV infection in asymptomatic HIV-positive patients presenting at two Opportunistic Infections Clinics (OIC) in two large urban hospitals in Harare.

## Methods

A cross-sectional study was performed on 152 consecutive patients (88 females; 64 males) attending OIC at Parirenyatwa Group of Hospitals and Harare Central Hospital between November 2014 and June 2015. Eleven patients opted not to be included in this study with the main reason given for refusal being lack of time. A total of 9000 adult patients are enrolled at the two OIC, with a male:female ratio of 1:1.5. The OIC serve a low to middle-income urban population of Harare, Zimbabwe. Patients visit the OIC for HIV testing and counselling services, routine follow up of HIV disease, and fulfilment of prescribed antiretroviral drugs. 85% of patients enrolled at the OIC are on highly active antiretroviral therapy (HAART). Ethical approval for this study was obtained from the University of Zimbabwe, College of Health Sciences Joint Research Ethics Committee and the Harare Central Hospital Ethics Committee. The study was registered with the Medical Research Council of Zimbabwe.

Males and females age ≥ 18 years were included in the study after providing informed signed consent. The source test document was checked to verify that each participant was HIV-positive. Demographic data and behavioural characteristics were collected through a face-to-face structured interview. CD4 counts and viral loads were verified by inspection of the participant’s clinical record.

### Anal sample collection

The participant was positioned on a standard examination couch in the left lateral position with knees drawn up to the chest and buttocks at the edge of the bed. The perianal region was inspectedafter gently parting the buttocks. A Dacron-tipped swab was moistened in 0.9% normal saline, then introduced blindly into the anal canal as far as possible [5]. Using the external anal sphincter as a fulcrum, the swab was firmly rotated against the anal canal wall for 30 s while being withdrawn from the anal canal. Clean scissors were used to cut off the Dacron tip of the swab into a labelled 2 ml cryotube containing 0.5 ml 10% guanidine thiocyanate and frozen at − 70 °C until analysis.

### HPV genotyping

HPV testing was done using primer pair MY09/MY11 polymerase chain reaction (PCR), followed by typing using the dot blot method, as described previously [5]. For internal quality control beta-globin was co-amplified during each PCR run and was probed for using a human beta-globin gene probe. Samples that tested negative for beta-globin were excluded from analysis.

Samples were thawed to room temperature. HPV DNA was extracted using the conventional ammonium acetate method. AmpliTaq Gold Polymerase enzyme was added to the samples to amplify target DNA. The PCR cycles were at: 95 °C for 9 min, 40 cycles of (95 °C for 1 min, 55 °C for 1 min and 72 °C for 1 min), 72 °C for 5 min and then held at 4 °C. After PCR, the DNA amplification mixture was denatured using an alkaline buffer then, applied to a Biodyne B membrane. The DNA was fixed to the Biodyne B membrane by baking at 80 °C for 1 h and then exposed to an HPV L1 consensus probe mixture. A sample was defined as HPV-positive if it reacted positively to the consensus probe mixture. Hybridisation was then carried out between the sample DNA and specific biotin-labelled HPV probes for 14 high-risk HPV genotypes (16, 18, 31, 33, 35, 39, 45, 51, 52, 56, 58, 59, 66 and 68) and 15 low-risk HPV genotypes (6, 11, 26, 32, 40, 53, 54, 55, 61, 69, 70, 73, 82, 83 and 84). The hybridization products were then detected using enhanced chemiluminescence (ECL). Commercial HPV standards were blotted onto each membrane as positive controls for their respective genotype.

### Statistical analysis

Data collection forms were inspected for completeness and accuracy by trained study personnel. Patients with missing data for a particular analysis were excluded only from that analysis. Data was analysed using IBM SPSS Statistics for Windows, version 22 (IBM Corp., Armonk, N.Y., USA). Comparison between groups of frequencies was performed with Chi-square test or Fisher’s exact test of association. All results were evaluated at a 95% confidence interval; significance was set at *p* < 0.05.

## Results

Data from 122 patients were analysed after excluding 30 whose samples tested negative for beta-globulin; 73 (60%) were female and 49 (40%) were male. The mean age was 39.6 years (Range 18–69; SD 10.7). All patients were Black Africans. A summary of the study population is shown in Table 1.

**Table 1:** Characteristics of the study population (*N* = 122)

Characteristic		
Age (years)	Mean (SD)	39.6 (10.7)
Sex		n (%)
	Male	49 (40.2)
	Female	73 (59.8)
Marital status		
	Single	21 (17.2)
	Married	67 (54.9)
	Divorced	13 (10.7)
	Widowed	21 (17.2)
Current CD4		
	Median (IQR)	375 (235–557)
CD4 nadir		
	Median (IQR)	141 (65–597)
Time since HIV diagnosis		
	< 1 year	15 (12.7)
	1 to 5 years	50 (42.4)
	> 5 years	53 (44.9)
Age at first sex		
	Median (IQR)	19 (18–21)
Number of lifetime sexual partners		
	< 5	69 (62.2)
	5 to 10	25 (22.5)
	> 10	17 (15.35)
Sexual orientation		
	Heterosexual	121 (99.1)
	Homosexual	1 (0.9)
Receptive anal intercourse		
	Ever	5 (4.1)
	Never	117 (95.9)
Condom use		
	Regular	71 (58.2)
	Occasional	44 (36.0)
	Never	7 (5.8)
History of anal warts		
	Yes	18 (14.8)
	No	104 (85.2)
History of CIN/cervical cancer (women only)		
	Yes	3 (4.1)
	No	70 (95.9)
History of VIN/vulvar cancer (women only)		
	Yes	0 (0.0)
	No	73 (100.0)

Whilst we set out to probe for 14 high-risk and 15 low-risk HPV genotypes, we chose not to report results for genotypes 26, 70 and 82 (intermediate risk) and genotypes 32, 54, 55 and 61 (low-risk) due to lack of availability of working standards for those particular genotypes. These genotypes were therefore excluded from analyses and are not reported. Fifty-four of 122 samples tested HPV positive (44%). Of the 54 HPV-positive samples, 49 (91%) had specific HPV genotypes detected. Anal HPV was detected in 10/49 males (20%) and in 44/73 females (60%). High risk HPV types were detected in 25 patients (20%); low-risk HPV types were detected in 24 patients (20%). Nine patients (7%) had multiple high-risk HPV types i.e. more than one specific high-risk HPV type detected per sample. All high-risk HPV genotypes that we sought except HPV-39 were detected in this study population.

Table 2 summarises the prevalence and type distribution of HPV genotypes in the study sample.

**Table 2:** Summary HPV prevalence by genotype

	Males		Females		All	
	(*n* = 49)		(*n* = 73)		(*n* = 122)	
	N	(%)	n	(%)	n	(%)
Any HPV type	10	20.4	44	60.3	54	44.3
Any low risk HPV type	5	10.2	19	26.0	24	19.7
Any high risk HPV type	5	10.2	20	27.4	25	20.5
Multiple high risk HPV types^a^	1	2.0	8	11.0	9	7.4

^a^More than one high-risk HPV genotype identified in a single patient sample

The most commonly detected high-risk HPV genotypes were HPV-58 (13%), HPV-31 (11%) and HPV-16 (9%). HPV-18 was detected in 2.5% of samples. HPV-11 (17%) and HPV-53 (11%) were the two most commonly detected low-risk genotypes.

**Fig. 1:**
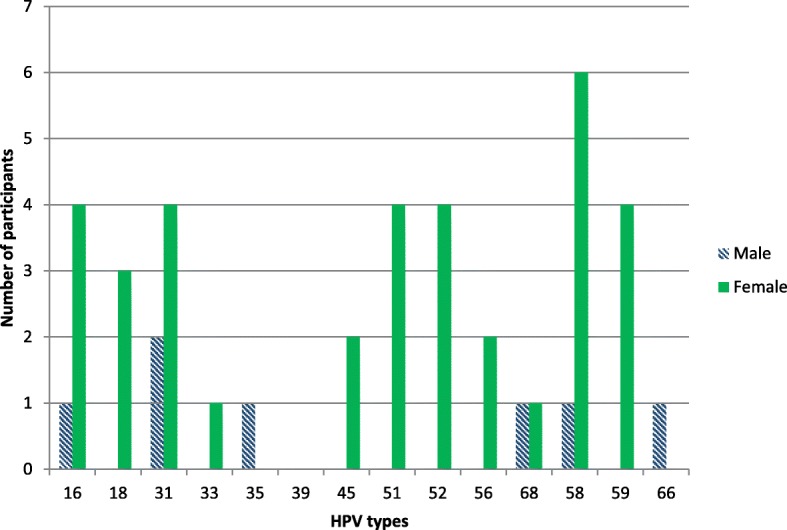
Distribution of high-risk HPV genotypes among patients who tested HPV positive

**Fig. 2:**
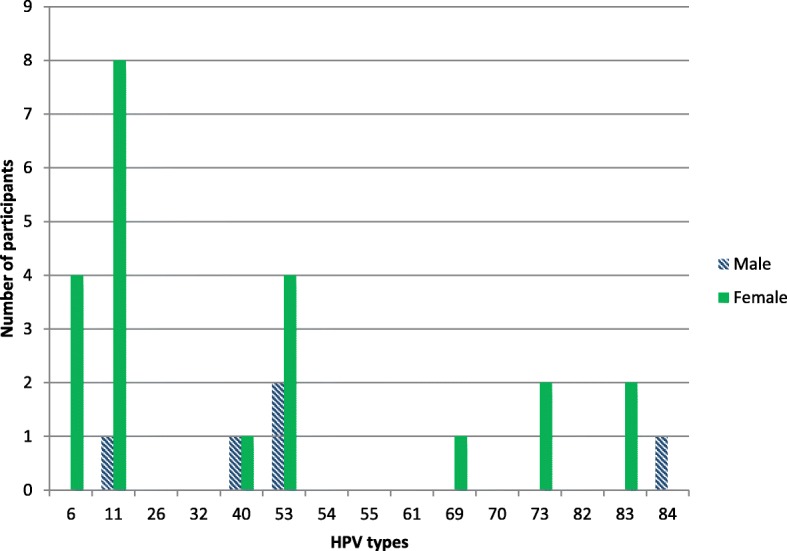
Distribution of low-risk HPV genotypes among patients who tested HPV positive. *Data for HPV-26, 32, 54, 55, 61, 70 and 82 are not reported (see text for detail)

Figures 1 and 2 show the type-specific distribution of anal HPV infections in the study sample.

A positive test for any HPV was significantly associated with female gender (*p* < 0.0001). It was not significantly associated with marital status, HAART (highly active anti-retroviral therapy) use, cigarette smoking, alcohol use, number of sex partners, condom use, current CD4 count nor nadir CD4 count. For males and females combined, detection of any high-risk HPV was significantly associated with ≥5 lifetime partners (*p* = 0.021). When data were analysed for males and females separately, there was no change noted in the above associations. A summary of statistical associations between a positive HPV result and selected socio-demographic and immunological factors is shown in Table 3.

**Table 3:** Relationship between positive HPV result and select demographic, immunological and behavioural variables

		Any HPV positive			Any high-risk HPV positive		
		(*N* = 54)			(*N* = 25)		
		n	%	*p*-value	N	%	*p*-value
Sex							
Male		10	20.4	0.000	5	11.6	0.294
Female		44	60.3		20	18.7	
Marital Status							
Single		8	44.4	0.552	3	15.0	0.634
Married		27	32.1		12	14.1	
Widowed		13	44.8		7	24.1	
Divorced		6	40.0		3	20.0	
Education							
Primary school		4	26.7	0.569	3	20.0	0.900
Secondary school		37	40.2		15	16.1	
Tertiary education		13	41.9		6	18.8	
Employment							
Formal employment		8	38.1	0.545	5	22.7	0.551
Informal employment		31	41.9		13	16.9	
Unemployed		15	31.9		6	12.5	
Currently on HAART							
No		2	33.3	0.809	0	0.0	0.256
Yes		52	38.2		25	17.7	
Time since HIV diagnosis							
< 1 yr		11	50.0	0.454	4	17.4	0.999
1 to 5 yr		24	38.7		11	17.7	
> 5 yr		19	34.5		10	17.5	
Smoking							
Current		1	33.3	0.991	0	0.0	0.643
Past		7	36.8		3	15.0	
Never		46	37.1		22	17.3	
Alcohol intake							
Current		4	57.1	0.534	1	12.5	0.751
Past		18	36.7		7	14.0	
Never		32	36.0		17	18.5	
Number of lifetime sex partners							
< 5		37	36.6	0.414	16	15.8	0.021
5 to 10		12	50.0		8	34.8	
> 10		5	41.7		0	0.0	
Condom use							
Regular		31	34.8	0.730	12	12.9	0.060
Occasional		7	36.8		3	15.8	
Never		6	46.2		5	38.5	
Sexual orientation							
Heterosexual		54	38.8		25	17.6	
Homosexual		0	0.0		0	0.0	

## Discussion

In this study of a largely heterosexual, HIV-positive population in Harare, Zimbabwe, the overall prevalence of anal HPV infection (high-risk and low-risk HPV genotypes) was 44%. This is the first paper describing prevalence of anal HPV infection in African heterosexual men, and one of the first in African women.

The anal HPV prevalence in females was three-fold higher than in men (60% vs. 20%). Most studies on females note that prevalence of anal HPV is actually higher than cervical HPV [6]. Putatively the cervix acts as a reservoir for HPV infections, allowing cross-infections with other sites such as the anus [7]. Simpson et al. suggest that in women, post-toilet front-to back wiping is a plausible biological mechanism for introduction of HPV into the anal canal [8]. There is likely under-reporting of anal sex in our study as this is a cultural taboo. Nonetheless, it is notable that the prevalence in males, though less than in females, is substantial, and suggests the importance of screening for anal cancer in both male and female HIV-positive patients regardless of self-reported sexual practices [9, 10].

High-risk HPV prevalence was 21% being found in 10% of males and 27% of females. Overall HPV-16 was found at a prevalence of 4% which is important because HPV-16 is one of the most consistent genotypes associated with anal cancer [11]. To date, however, studies on prevalence of HPV-16 in anal cancer samples have included no or very few samples, from Africa. In a recent meta-analysis by Lin et al. that included 2358 anal cancer samples, only 23 samples were from studies in Africa [12]. As more data become available it will become clearer how significant a role HPV-16 plays in anal cancer within the African population. An interesting finding in our data is that HPV-58 was the most common high-risk type detected. Other local and regional studies have also noted HPV-58 to be amongst the most frequently detected in the genital region [13, 14]. This may reflect different geographical distribution of HPV genotypes than reported elsewhere in the world. In addition, or alternatively, the frequent detection of HPV-58 may be a reflection of the relatively slow rate at which this particular genotype is cleared. Shvetsov et al. found that HPV-59 and 58 had the longest clearance times in a Hawaiian study of healthy women [15].

Overall, low-risk HPV infections were as common as high-risk infections in our study (20% vs. 21%). Though low-grade types are much less oncogenic by definition, they cause a significant burden of genital warts. The majority of these warts are attributable to infection by HPV-6 and 11 [16]. These are bothersome to the patient and difficult to treat [17]. This is an important consideration as Zimbabwe, through the Ministry of Health and Child Care, is in the process of rolling out a nationwide vaccination programme. The currently available vaccine is divalent, providing protection against HPV-16 and HPV-18 [18]. Ideally a vaccine protecting against HPV-6 and HPV-11 would reduce the future burden of genital warts.

There are several limitations to our results. The study was cross-sectional yet many HPV infections are transient [15]. We used the MY09/MY11 primer set for PCR and the dot blot method for HPV typing. These are older methods compared to newer technologies such as next-generation DNA sequencing. They are not able to detect all possible HPV types in the anogenital region but enabled us to describe the prevalence of the most important HPV types known to be associated with cervical and anal cancer and their precursor lesions. In addition we chose not to report on the prevalence of 7 other lower risk HPV genotypes (26, 32, 54, 55, 61, 70 and 82). Our overall HPV positivity remains unaffected because consensus primers were used initially, as do our results for the specific genotypes most commonly associated with anogenital cancer in Africa. Self-reporting of sexual practices may be unreliable especially where such practices are stigmatising. There is likely under-reporting of anal sexual intercourse in our study, especially by men, for this reason. Our study was set in two urban hospital clinics. This limits the generalisation of our findings to the rest of Zimbabwe where behavioural risk for sexually transmitted infections and access to health care may be different.

## Conclusions

In conclusion the prevalence of anal HPV was 44% in this group of largely heterosexual HIV-positive males and females. Oncogenic HPV genotypes accounted for almost half of infections, supporting the need for surveillance of anal cancer in this population. These data represent a baseline upon which future studies may be built. Specifically, longitudinal studies can potentially reveal the pattern of persistence of anal HPV and chronicle the development of cytopathological changes over time.

## Abbreviations

DNA: Deoxyribonucleic acid
HAART: Highly active antiretroviral therapy
HIV: Human immunodeficiency virus
HPV: Human papillomavirus
MSM: Men who have sex with men
OIC: Opportunistic Infections Clinic
PCR: Polymerase chain reaction
